# Improving breastfeeding among adolescent mothers: a prospective cohort

**DOI:** 10.1590/1516-3180.2022.0647.R1.260723

**Published:** 2023-11-20

**Authors:** Maira Pinho-Pompeu, Renan Massao Nakamura, Erika Zambrano, Fernanda Garanhani Surita

**Affiliations:** I PhD. Nutritionist, Departamento de Tocoginecologia, Faculdade de Ciências Médicas, Universidade Estadual de Campinas (UNICAMP), Campinas (SP), Brazil.; II MD. Resident Physician, Departamento de Tocoginecologia, Faculdade de Ciências Médicas Universidade Estadual de Campinas (UNICAMP), Campinas (SP), Brazil.; III PhD. Obstetric nurse, Assistent Professor, Faculdade de Enfermagem, Universidade Estadual de Campinas (UNICAMP), Campinas (SP), Brazil.; IV MD, PhD. Obstetrician, Full Professor of Obstetrics, Departamento de Tocoginecologia, Faculdade de Ciências Médicas, Universidade Estadual de Campinas (UNICAMP), Campinas (SP), Brazil.

**Keywords:** Breast feeding, Prenatal care, Prenatal education, Pregnancy in adolescence, Postpartum period, Antenatal parenthood education, Breastfeeding, Teen pregnancy

## Abstract

**BACKGROUND:**

Exclusive breastfeeding is recommended for the first six months, and mother’s age impact early weaning. Educational support and relevant information can increase breastfeeding rates.

**OBJECTIVE:**

To determine whether antenatal education enhances the maintenance, intention, and confidence in breastfeeding among adolescents.

**DESIGN AND SETTING:**

A prospective cohort study involving primiparous adolescents who gave birth at the Woman’s Hospital (CAISM), Universidade Estadual de Campinas, Brazil.

**METHODS:**

Adolescent mothers were categorized into two groups based on the location of prenatal care: those at the Woman’s Hospital (WH) who received antenatal education, and at the Primary Care (PC) who did not receive antenatal education. All adolescents received breastfeeding orientation during their postpartum hospital stay. The groups were compared using the Student’s t-test, Mann–Whitney U test, and chi-squared test. Log-binomial models were used to compare the groups at different time intervals.

**RESULTS:**

The study included 132 adolescents: 59 in the WH group and 73 in the PC group. Six months postpartum, adolescents in the WH group demonstrated higher engagement in breastfeeding (P < 0.005) and exclusive breastfeeding (P = 0.04) than PC group. PC group showed greater lack of confidence in breastfeeding (P = 0.02) and felt less prepared (P = 0.01). Notably, all WH adolescents reported a stronger desire to breastfeed after antenatal education.

**CONCLUSION:**

Antenatal education significantly improves the maintenance, intention, and confidence of breastfeeding among adolescents. This education approach can be implemented across all healthcare levels and should be made accessible to all women throughout the pregnancy and postpartum period.

## INTRODUCTION

Breastfeeding, due to its protective benefits for the mother and its role in promoting optimal child development, is advocated as the sole form of neonatal nourishment during the initial 6 months of life. It is recommended to continue until the child reaches 2 years of age and beyond.^1^ Breastfeeding women are less likely to develop breast and ovarian cancer, type 2 diabetes, postnatal depression, and osteoporosis.^2,3^ Additionally, infants nourished with human milk exhibit enhanced protection against infections, asthma, leukemia, and sudden infant death syndrome.^3^ Neonatal breastfeeding also has a long-term effect on reducing the prevalence of obesity, heart disease, and diabetes.^3,4^ Regrettably, the global breastfeeding rate falls short of the ideal benchmark. Only 42.0% of infants worldwide are exclusively breastfed during their first 6 months postpartum.^5^ Alarmingly, breastfeeding rates decline in correlation with income or education status, particularly in low- and middle-income countries.^3^

The age of a mother significantly contributes to the low rates of breastfeeding, with the challenges of motherhood being particularly amplified during adolescence. When compared to women aged 20–29 (36.4%) and those over 30 (45.0%), adolescent mothers are the least likely to exclusively breastfeed their newborns in the first 6 months, with rates falling below 25.0%. Social and cultural norms predominantly influence the decisions of adolescent mothers not to breastfeed.^5,6^

Data on exclusive breastfeeding rates among adolescent mothers is limited. Studies from Brazil have noted a steady decrease in exclusive breastfeeding during the first 6 months postpartum among this demographic. The authors propose that maternal age is not the sole factor linked to early cessation of breastfeeding, suggesting that teenage motherhood possesses distinct attributes.^7^ Adolescents often encounter conflicting situations during this period, potentially leading to feelings of psychological incapacity. Given that pregnancy itself is a vulnerable situation, the state of motherhood can induce feelings of insecurity, anxiety, and fear. These emotional changes may jeopardize breastfeeding practices, causing these young mothers to breastfeed their children for a shorter duration than recommended by the WHO. Furthermore, they may lack understanding or information about the importance of breastfeeding for their child’s development.^7^

A woman’s understanding of the significance and management of breastfeeding is a crucial factor associated with early weaning. A study involving 297 women demonstrated that knowledge about breastfeeding influenced the choice of child-feeding method (breast milk and/or infant formula) and the duration of breastfeeding.^8^ Furthermore, a study on breastfeeding self-efficacy among teenage mothers revealed that 56.90% exhibited a high level of self-efficacy, 35% showed a moderate level, and 8.10% had a low level. These results suggest that adolescents with high breastfeeding self-efficacy tend to breastfeed exclusively for a longer period.^9^ Family members, prenatal care professionals, and the media serve as the primary sources of breastfeeding information for teenage mothers.^7^ Consequently, it is crucial for healthcare professionals to offer additional support to teenagers during the postpartum period, fostering a more enjoyable and lasting breastfeeding experience.^9^

Despite the existence of laws advocating for breastfeeding and the presence of an extensive and intricate network of milk banks, Brazil continues to exhibit a low rate of exclusive breastfeeding among infants aged 6 months or less (36.6%). This rate falls short of the Global Nutrition Target 2025, which is set at 50.0%. Notably, the mother’s age plays a crucial role in early weaning.^10–12^

The global teenage pregnancy rate is estimated at 46 births per 1,000 girls, constituting a significant public health concern, particularly in low and middle-income countries.^13^ Various interventions, either standalone or combined, have been employed to enhance the initiation or prolongation of breastfeeding among mothers. These interventions encompass social, physical, and educational support, the latter offering women vital information about breastfeeding.^4^

The primary objective of this study was to compare the 6-month postpartum breastfeeding rates between adolescents who received antenatal breastfeeding education and those who did not. The secondary objectives were to examine the impact of antenatal education on a mother’s confidence in breastfeeding and her intention to exclusively breastfeed.

## METHODS

### Design

We conducted a prospective cohort study involving primiparous adolescents at the Woman’s Hospital, University of Campinas, Campinas, Brazil. This hospital is a referral center for high-risk obstetrics, offering specialized antenatal care for pregnant teenagers through an interdisciplinary, multi-professional team.

### Characteristics of the sample

All primiparous adolescents aged 19 or under who delivered a single, live infant at the Women’s Hospital were chosen for the study. Their medical records were examined to divide the adolescents into two categories: those who received prenatal care at the Women’s Hospital and those whose pregnancies were overseen at primary healthcare facilities. After this initial categorization, all adolescents were queried about whether they received breastfeeding guidance during prenatal care, as the study’s objective was to comprehend the impact of antenatal education on breastfeeding. Subsequently, adolescents were invited to participate in the study and were divided into two groups:

- Adolescents who received prenatal care and breastfeeding guidance at the Woman’s Hospital;
- Adolescents who received prenatal care in primary healthcare facilities but did not receive guidance on breastfeeding.

The study excluded adolescents who received prenatal care at the Woman’s Hospital without obtaining breastfeeding guidance, as well as those whose pregnancies were managed in primary healthcare facilities but did receive breastfeeding instruction.

The exclusion criteria encompassed primiparous adolescents with newborns diagnosed with malformations and/or requiring intensive care, those diagnosed with human immunodeficiency virus, those prescribed medication incompatible with breastfeeding, those with psychiatric disorders, and those with hearing or cognitive deficiencies.

### Antenatal education

Since 2003, the Woman’s Hospital has held accreditation from the Baby Friendly Hospital Initiative (BFHI).^14^ In line with BFHI’s recommendations, trained nursing staff provide group orientation on breastfeeding to all pregnant women receiving antenatal care. Additionally, the Woman’s Hospital consistently offers breastfeeding orientation and support throughout labor and the postpartum hospital stay. To uphold the ten steps to successful breastfeeding and ensure consistent quality, all healthcare professionals involved in promoting and supporting breastfeeding undergo BFHI training and certification. This guarantees that all accredited healthcare facilities maintain the same high standards.^14^

The outpatient clinic routinely offers an open antenatal education group for pregnant teenagers, focusing on various themes related to adolescent pregnancy. This group provides a secure environment and aims to empower these young women through educational interventions. Topics covered include sexual and reproductive rights, contraception, mental health, newborn care, health awareness, and gender issues. The group convenes twice weekly, during both antenatal and postpartum care periods for adolescents.

### Data collection

The adolescents were categorized into two groups based on their antenatal care location. The Woman’s Hospital (WH) group consisted of adolescents who received pregnancy monitoring at the hospital, thus having access to antenatal education programs. The Primary Care (PC) group comprised adolescents monitored in primary healthcare facilities. Data collection from each participant occurred at three intervals: within 1–3 days post-childbirth (during the postpartum hospital stay), 40–60 days post-childbirth (during the 1^st^ postpartum care visit), and 6 months post-childbirth.

The initial time point takes place in the rooming-in setting, a designated area for accommodating the mother-baby dyad during the postpartum hospital stay. This setting is staffed by a multi-professional team available 24 hours a day to assist women and newborns without perinatal or delivery complications. Consequently, dyads with a contraindication to breastfeeding, such as severe prematurity, are not allocated to the rooming-in setting.

Data about breastfeeding intent and confidence were gathered during the postpartum hospital stay. At the initial postpartum care visit, participants completed a questionnaire regarding breastfeeding maintenance, the newborn support network at home, and pacifier use. Six months post-childbirth, a follow-up phone interview was conducted with the adolescent mothers, during which they were once again asked about breastfeeding maintenance.

The authors designed a questionnaire to assess participants’ confidence in breastfeeding, posing the following closed-ended questions: “During pregnancy, were you prepared to breastfeed your child?” and “Upon first holding your child to breastfeed, did you know how to proceed?” To gauge participants’ intent to breastfeed, the question asked was: “Did your participation in the WH influence your decision to breastfeed your child?”

The secondary outcomes included: the primary subjects remembered by WH adolescents from the antenatal education (e.g., “Can you recall the topics discussed during the antenatal education?”); the source of breastfeeding information during pregnancy among PC adolescents; the influence of a mother’s primary support network; and the utilization of a baby pacifier.

During the postpartum hospitalization, sociodemographic, obstetric, and perinatal outcomes were gathered from both the medical record and the prenatal card.

### Sample size

The study’s sample size was determined with the aim of comparing exclusive breastfeeding rates 6 months postpartum. However, comparisons were also made during the initial postpartum visit and across two distinct periods within each group, resulting in four comparison groups. The sample size calculation was based on the methodology for a Pearson’s Chi-square test,^15^ with a significance level of 1.25%, a test power of 80.0%, and an assumed effect size of 0.30, which is considered a medium effect size.^16^ Consequently, a minimum of 124 participants was required for the study.

### Statistical methods

Descriptive analysis was conducted using the mean and standard deviation for numerical variables, and percentage and n for categorical variables. Bivariate analyses, including Student’s t-test, Mann–Whitney, and chi-squared tests, were utilized to compare the groups. Log-binomial models were also calculated to compare the groups across different periods when breastfeeding rates were observed. The level of significance was set at 5%. Stata 17 version 14.0 for Windows (64 bit) (StataCorp, College Station, United States) was the statistical software employed. To ensure data accuracy, double-typing was executed using Microsoft Excel software for Windows (Microsoft, Redmond, United States).

### Ethics

The Ethics and Research Committee of UNICAMP approved this study on July 20, 2017 (CAAE: 69198417.4.0000.5404; number: 2.180.783, date: July 20, 2017). All participants under the age of 18, after reading, understanding, and having their queries addressed, signed an informed consent form, which was countersigned by their legal representative. Participants aged 18 years and older provided their signatures on the consent form. The study procedures strictly adhered to the STROBE guidelines.^17^

## RESULTS

Between August 2018 and February 2019, 132 adolescents were included in the study, with a mean age of 16.7 (± 1.2) years (Table 1).

**Table 1:** Sociodemographic and anthropometric characteristics, number of antenatal care visits, and perinatal outcomes of adolescent mothers (n = 132)

	Woman’s Hospital (n = 59)		Primary Care (n = 73)		P value
	Mean	Standard deviation	Mean	Standard deviation	
Age (years)	16.2	1.3	16.9	1.12	0.01^b^
Number of antenatal care visits	10.6	2.7	9.2	2.2	0.03^b^
BMI before pregnancy	22.7	5.3	23.4	5.5	0.63^b^
Gestational weight gain	10.6	6.7	12.1	6.9	0.27^b^
Newborn weight (g)	3127.7	367.6	3009.9	404.8	0.16^a^
	n	%	n	%	P value
White skin color	25	64.1	26	53.1	0.58^c^
With partner	30	81.1	32	68.1	0.18^c^
Student	26	70.2	26	53.1	0.11^c^
Compatible age-degrees	27	77.1	37	77.1	0.99^c^
Gestational age < 37 (weeks)	1	1.8	7	10.3	0.07^c^
Vaginal delivery	26	70.3	38	79.2	0.35^c^
Newborn weight < 2.500g	4	6.9	5	7.1	< 0.99^c^

a Student’s t-test;

b Mann–Whitney test;

c Chi-squared test.

**Figure 1:**
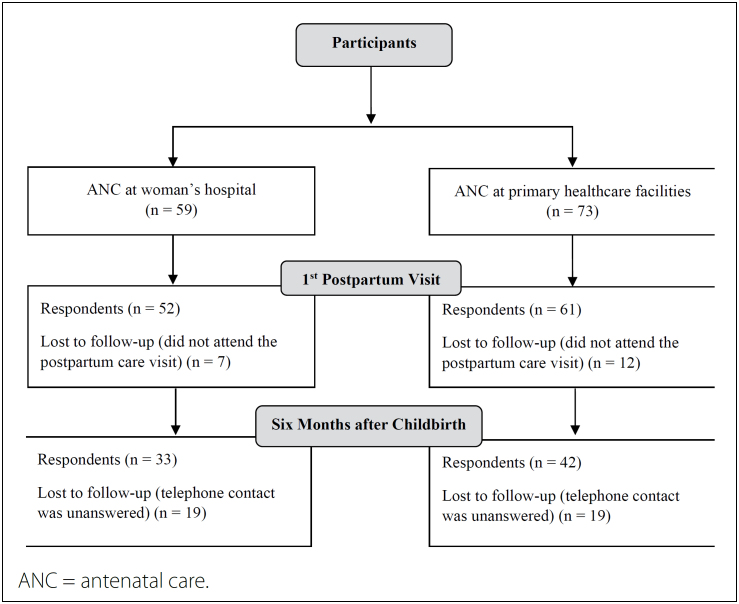
Flowchart of participants’ progress through the points of the cohort.

Following the distribution, 59 adolescents were allocated to the WH group, while 73 were assigned to the PC group (Figure 1). In the WH group, 11.9% (7) of the girls failed to attend the initial postpartum visit, compared to 16.4% (12) in the PC group (P = 0.46). Six months post-childbirth, 36.5% (19) of the adolescents in the WH group and 31.1% (19) in the PC group did not respond to the telephone call (P = 0.54).

Of the WH participants, six (15.8%) reported feeling unprepared to breastfeed their children, compared to 21 (43.7%) of the PC participants (P = 0.01). Furthermore, 12 (31.6%) of the WH participants and 29 (56.8%) of the PC participants were unsure about how to breastfeed when they held their newborns for the first time (P = 0.02).

At the first postpartum care visit, 74 (65.5%) of all adolescents were breastfeeding their children, 50 (44.2%) of them exclusively. At 6 months following childbirth, 60 (80%) of all adolescents were breastfeeding their children, 31 (41.6%) of them exclusively. The rates of breastfeeding by group and over time are described in Table 2 and Table 3, respectively.

**Table 2:** Univariate logistic regression on maintenance of breastfeeding among primiparous adolescents according to the place where antenatal care was given (n = 132)

		Woman’s Hospital		Primary Care		OR	95% CI^a^	P value
		n/total	%	n/total	%			
**Breastfeeding**								
	Postpartum care visit	38/52	97.4	36/33	70.6	1.38	1.15–1.66	> 0.001
	Six months after delivery	32/61	82.5	28/42	54.9	1.49	1.12–1.99	0.006
**Exclusive Breastfeeding**								
	Postpartum care visit	26/52	66.6	24/33	47.1	1.42	0.98–2.04	0.062
	Six months after delivery	18/61	46.1	13/42	25.5	1.81	1.01–3.23	0.044x

OR = Odds ratio; CI = confidence interval;

a 95%CI OR = 95% confidence interval for odds ratio.

**Table 3:** Univariate logistic regression on the maintenance of breastfeeding over time among primiparous adolescents (n = 132)

		Postpartum care visit		Six months after delivery		OR	95% CI^a^	P value
		n/total	%	n/total	%			
**Breastfeeding**								
	Woman’s Hospital	38/52	97.4	32/33	82.0	0.84	0.72-0.99	0.034
	Primary Care	36/61	70.6	28/42	54.9	0.78	0.65-0.93	0.005
**Exclusive Breastfeeding**								
	Woman’s Hospital	26/52	66.7	18/33	46.1	0.69	0.54-0.89	0.005
	Primary Care	24/61	47.1	13/42	25.5	0.54	0.36-0.81	0.003

OR = Odds ratio; CI = confidence interval;

a 95% CI OR = 95% confidence interval for odds ratio.

All WH participants indicated that participating in antenatal education increased their intent to breastfeed their children. Recollection of the topics covered during the antenatal education resulted in the following: the advantages of breastfeeding (n = 41; 71.9%); the importance of breastfeeding in the first hour of a child’s life (n = 29; 50.9%); and how to take care of one’s breasts during breastfeeding (n = 24; 42.1%).

Among PC participants, 47 (64.4%) did not receive information about breastfeeding during pregnancy and described only having received such information during their postpartum hospitalization at the Woman’s Hospital. The main sources of breastfeeding information for adolescents with PC were healthcare professionals (n = 12; 16.4%), family members (n = 11; 15.1%), and the internet (n = 10; 13.7%).

The adolescents consistently identified their primary support network across both groups: their partner (if present) and their mother. The use of a baby pacifier was noted in 15 (39.5%) of the WH participants and 32 (62.7%) of the PC participants (P = 0.03).

## DISCUSSION

This research demonstrates that antenatal education positively impacts adolescents’ ability to sustain breastfeeding for the first 6 months post-childbirth. It also positively affects a mother’s intention to breastfeed. In general, adolescent mothers reported feeling more prepared to breastfeed after participating in antenatal education.

The observed rate of exclusive breastfeeding at 6 months postpartum (41.6%) exceeded the rate reported in the literature for teenagers.^6,18^ However, this rate falls short of the global rate (42%) ^5^ and is significantly lower than the Global Nutrition Targets 2025 (50%).^10^ In our study, adolescents from WH breastfed for a longer duration than those from PC, regardless of exclusivity. Other studies that explored the impact of education and support provided to breastfeeding mothers have also noted a positive effect of antenatal education on breastfeeding, not just among adolescents but also in the adult female population.^3,4,18,19^ Moreover, research has shown that breastfeeding education can positively influence breastfeeding practices even when offered solely during the postpartum hospital stay and/or the breastfeeding period.^4,20,21^ In our study, all adolescents, both from WH and PC, received breastfeeding education during their postpartum hospital stay. This could account for the higher breastfeeding rate achieved in comparison to the rates reported in the literature.^6,20^

A notable decline in breastfeeding rates was observed six months postpartum, even among WH participants. This finding underscores the necessity of not just educating mothers about breastfeeding but also providing sustained social support, especially for adolescent mothers. A qualitative study involving young mothers identified four primary obstacles to breastfeeding: stigma, role, place, and support. Stigma relates to the embarrassment of breastfeeding in public and the identity of being a young mother. Role refers to the difficulties of juggling the dual responsibilities of being an employee or student and a mother. The place barrier involves the lack of time or support at school or work, coupled with the absence of facilities to store expressed milk. Lastly, the support barrier is tied to the lack of adequate breastfeeding support within the broader community or from unsupportive family members.^5,21^

Participants in the WH were more adequately prepared to breastfeed their infants upon first holding them. Additionally, adolescent participants in the WH reported an increased intention to breastfeed following their involvement in the antenatal education group. Other research involving both adolescent and adult mothers has suggested that frequent attendance at support group meetings leads to improved attitudes toward breastfeeding, reduced barriers to breastfeeding, and increased breastfeeding rates.^3,20,22^

Emphasizing the significance of a higher breastfeeding rate is crucial, as it contributes to the attainment of the Sustainable Development Goals (SDG.) Research has demonstrated that breastfeeding can enhance educational achievement and income in adulthood, thereby addressing SDG1: no poverty, SDG4: quality education, and SDG8: decent work and economic growth. Furthermore, breastfeeding can help prevent hunger, malnutrition, and obesity, aligning with SDG2: zero hunger and SDG3: good health and well-being.^23^ Additionally, the right of women to breastfeed and express milk in public spaces is recognized, supporting SDG5: gender equality.

Our observation revealed that a significant proportion (64.4%) of PC adolescents did not receive any information about breastfeeding during antenatal care. The majority of these adolescents obtained breastfeeding information from their family and friends. However, health professionals are deemed the most qualified individuals to provide adolescents with breastfeeding advice. The internet was another significant source of breastfeeding information reported. It is crucial to underscore that the participants in our study are adolescents from “Generation Z.” Consequently, the internet and social media play a substantial role in their lives and can also serve as a valuable platform for healthcare professionals and organizations to advocate for exclusive breastfeeding practices.^24^

We observed a minor, albeit insignificant, difference in the prematurity rate between adolescents in the PC group and those in the WH group. Prematurity often poses a significant challenge to successful breastfeeding due to the increased suckling difficulties experienced by premature infants.^25^ At the Women’s Hospital, all mother-infant pairs in the rooming-in setting have the opportunity to breastfeed. Premature infants who are unable to breastfeed are accommodated in the Neonatal Care Unit. Therefore, in our study, prematurity was not deemed a source of bias.

Both groups included adolescent mothers with partners. The literature extensively documents the beneficial impact of a father’s presence on a child and the breastfeeding regimen.^18,26^ Regular interaction, such as cohabitation, with grandmothers, has been linked to a decrease in breastfeeding initiation and an increased risk of early weaning. Conversely, support from maternal grandmothers for breastfeeding has a positive correlation with the maintenance of breastfeeding.^18^

The adolescent demographic in WH was slightly younger compared to that in PC. This could be attributed to WH being a tertiary referral hospital, offering specialized antenatal care for teenagers. A notable difference between the two groups was the number of ANC visits. Nevertheless, the number of antenatal visits in both groups adhered to the WHO recommendation of eight health visits for pregnant women.^27^

Our study is subject to certain limitations. Primarily, the healthcare professionals disseminating information to the multidisciplinary and BFHI groups could vary on a weekly basis. Nevertheless, all healthcare professionals involved in breastfeeding promotion and support have undergone BFHI training and certification, independent of this study. This is to mitigate potential discrepancies in guidance within the group and to ensure uniformity in the approaches to the topics discussed. Secondly, akin to other studies,^28^ a substantial number of missed follow-up appointments were noted. Factors such as sociodemographic, cultural, and logistical determinants could potentially contribute to higher rates of missed follow-ups among adolescents. To counteract this, adolescents who missed their postpartum care visit were promptly contacted via telephone to reschedule. Lastly, the retrospective questions posed during the hospital stay may have induced recall bias, as the adolescent mothers were physically exhausted and preoccupied with newborn care.

## CONCLUSIONS

We advocate for all expectant women, particularly adolescent ones, to receive antenatal education on breastfeeding to boost breastfeeding rates. When group participation is impractical, it falls to healthcare professionals to guide and support expectant and postpartum mothers through their breastfeeding journey. Antenatal education groups, being cost-effective and capable of accommodating a larger number of women, can act as a catalyst in low- and middle-income countries.
